# The clinical characteristics and the features of immunophenotype of peripheral lymphocytes of adult onset chronic active Epstein-Barr virus disease at a Tertiary Care Hospital in Beijing

**DOI:** 10.1097/MD.0000000000009854

**Published:** 2018-03-02

**Authors:** Ling Luo, Huanling Wang, Hongwei Fan, Jing Xie, Zhifeng Qiu, Taisheng Li

**Affiliations:** Department of Infectious Diseases, Peking Union Medical College Hospital, Chinese Academy of Medical Sciences, Beijing, China.

**Keywords:** adult onset, CAEBV infection, immunophenotype

## Abstract

Chronic active Epstein-Barr virus (CAEBV) infection is a rare disease with high mortality. Most of CAEBV patients have been reported from Japan and are pediatric cases.

The goal was to describe the clinical characteristics and the immunophenotypic features of peripheral lymphocytes in adult onset CAEBV patients.

We retrospectively reviewed and analyzed all adult onset CAEBV cases admitted to Peking Union Medical College Hospital (PUMCH) between 2012 and 2016. Demographic, clinical, laboratory data, and the immunophentyping data of peripheral lymphocytes were collected.

There were 28 adult onset CAEBV patients. The median age was 45 (range, 20–81). Most of the patients presented with fever; splenomegaly; lymphadenopathy and hepatitis. Unlike pediatric cases reported, the manifestations of cardiovascular diseases in our patients were pulmonary arterial hypertension, decreased cardiac function and aorta vasculitis. Prevalence of interstitial pneumonitis in our patients were comparatively higher and prevalence of hypersensitivity to mosquito bites were comparatively lower than that reported by Japan. In this study, CAEBV patients had decreased B cell, NK cell, CD4 cell and CD8 cell counts. The prevalence of low level of B cells, NK cells, CD4 cells was relatively higher than reported ever.

Chinese adult onset CAEBV patients have different clinical characteristics and are featured by an immunosuppression status as demonstrated by decreased B cell, NK cell, CD4 cell and CD8 cell.

## 1 Introduction

Epstein-Barr virus (EBV), also called human herpesvirus 4, is a ubiquitous DNA virus belonging to the gamma subfamily of herpesviruses. About 95% of adults are infected with EBV and the infection persists for life.^[1]^ Although most infections are asymptomatic, infection in adolescents or young adults often results in infectious mononucleosis (IM). Infectious mononucleosis often presents with fever, pharyngitis, lymphadenopathy, and splenomegaly, and these symptoms resolve without sequelae in most cases.^[2]^ In rare cases infected with EBV, these complications persist and progress and often developed a life-threatening condition.^[3–5]^ These patients are termed chronic active EBV disease (CAEBV). CAEBV infection is a rare disease with high mortality. It is a lymphoproliferative disorder characterized by elevated levels of EBV DNA in the blood and EBV RNA or protein in lymphocytes in tissues.

The majority of clinical cases with CAEBV were reported in Japan. Most of CAEBV patients reported were children or adolescents whose age were less than 12 years old.^[2,6]^ Study on the clinical characteristic of adult onset CAEBV from China are rare. The pathogenesis and pathophysiology of CAEBV are not well characterized. In primary infection, the response of CD8+ cell is crucial to control the infection, and these cells probably give rise to most of the symptoms and signs of IM as a result of high numbers CD8+cells in circulation and tissues and massive production of cytokines. That CD8+ cells are very important for recovery from IM is exemplified by the consequence of immunocompromised primary EBV-infected patients who are unable to mount the appropriate CD8+ T cell response. They usually die of a fulminating IM-like syndrome. This is the case of children affected by the X-linked lymphoproliferative syndrome (XLPS). However, the amount and levels of activation of CD8+ cell in CAEBV patients remained unknown especially in adult onset cases. In addition, the status of cell immune of CAEBV patients are not well characterized. Cohen et al. found abnormal proportions of lymphocyte subsets in most of the CAEBV patients from the United States.^[7]^ Study on the immunophenotypic features of peripheral lymphocytes in adult onset CAEBV patients are very limited.

Our goal was to retrospectively review adult onset CAEBV cases at Peking Union Medical College Hospital (PUMCH) and describe the clinical characteristics and the immunophenotypic features of peripheral lymphocytes of the patients.

## 2 Materials and methods

We performed a computer-assisted search and retrospectively identified adult onset CAEBV at the Peking Union Medical College Hospital (PUMCH) between 2012 and 2016.

CAEBV was defined according to proposed diagnostic guidelines as follows: (1) a severe progressive illness of > 6-month duration usually with fever, lymphadenopathy, and splenomegaly that either began as a primary EBV infection or was associated with high titers of IgG antibody to EBV-VCA and with little or on antibody to EBNA, or markedly elevated EBV DNA in the blood; (2) elevated EBV DNA, RNA, or proteins in affected tissues; and (3) the absence of any other immunosuppressive condition.^[8]^

The adult onset cases diagnosed as chronic active EBV were selected with the exclusion of leukemia or lymphoma and exclusion the CAEBV patient whose age was less than 18 years old. All the results for patients examined were negative for human immunodeficiency virus antibody.

Clinical data from the identified cases were abstracted from the medical records. These data included demographic characteristics, age of admission, symptoms, physical examination, complications, laboratory data at diagnosis, EBV-specific antibodies, virus loads were collected and analyzed. In situ hybridization for EBV-encoded mRNA (EBER) was performed on fixed paraffin-embedded sections with the use of an EBER1 riboprobe. Hemophagocytic syndrome (HPS) was defined according to proposed diagnostic guideline.^[9]^

Flow cytometric immunophenotyping was performed as previously described.^[10,11]^ In brief, whole blood was incubated with monoclonal antibodies against CD3, CD4, CD8, CD19, CD16, CD56, CD28, CD38 and HLA-DR (Beckman-Coulter, Brea, California, USA). Absolute counts of CD3+CD4+ cells, CD3+CD8+ cells were then calculated using the results from the complete blood cell and lymphocyte differential counts.

We used descriptive statistics. Continuous variables were summarized using median and range and categorical variables were summarized with percentage. This study was reviewed and approved by the Institutional Review Board at PUMCH. The study was conducted in accordance with the guidelines of the Declaration of Helsinki.

## 3 Results

A total of 28 cases were identified, and their clinical features were summarized in Table 1. There were 12 males and 16 females with a median age of 45 years old (range 20–81). The most common symptoms and signs were fever in 100% of patients, splenomegaly in 89.2%, lymphadenopathy in 85.7%, hepatitis in 60.7%, and hepatomegaly in 42.8%.

**Table 1:**
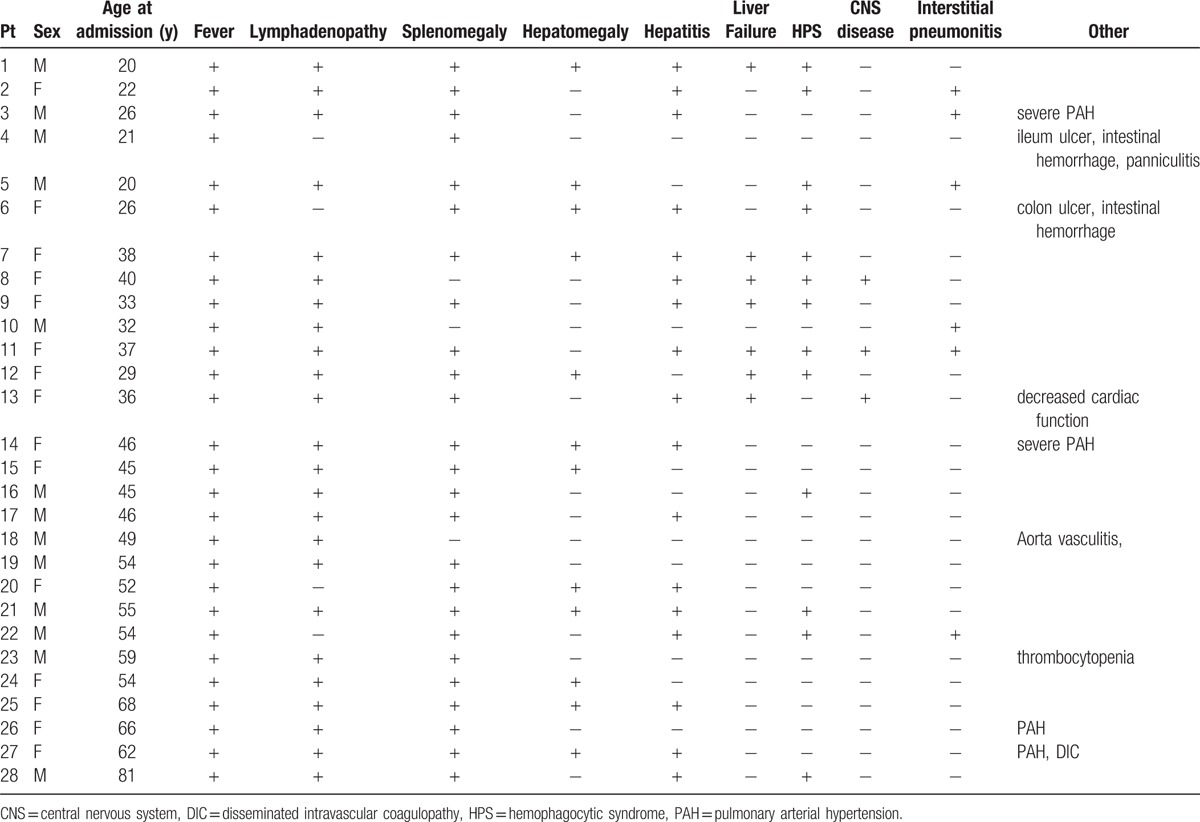
Clinical findings of CAEBV.

Cardiovascular diseases developed in these CAEBV patients included pulmonary arterial hypertension (4 cases, 14.2%), decreased cardiac function (1 case, 3.6%) and aorta vasculitis (2 case, 3.6%). Interstitial pneumonitis was observed in 25% of these CAEBV patients. 10.7% of these patients had central nervous system involvement and 7.1% had intestinal ulcers. No patient with hypersensitivity to mosquito bites (a finding usually associated with NK cell CAEBV) was found.

Life-threatening complications including HPS (16 cases, 57.1%), liver failure (7 cases, 25%), severe pulmonary arterial hypertension (PAH, 2 cases, 7.1%), intestinal hemorrhage (2 cases,7.1%), disseminated intravascular coagulopathy (1 case, 3.6%) were observed.

The median of white blood cell count is 3.23 × 10^9^/L (range 0.27–50.42 × 10^9^/L). 17 cases (60.7%) had anemia. Pancytopenia were noted in 9 cases, 8 of them were associated with HPS. 17 cases had abnormal liver function tests (>2 upper limit of normal, ULN). A reduction in serum albumin was observed in 75% of the cases. 20 cases (71.4%) had elevated lactate dehydrogenase levels. The median of Ferritin of 1809 (range 249–19456). The median of erythrocyte sedimentation rate is 18 mm/h (5–125). The median of high sensitive C reactive protein is 22.45 mg/L (range 4.23–61.55).

Viral load was measured in all the 28 cases using quantitative PCR. All cases showed increased EBV DNA copy numbers in the serum. The median serum EBV DNA with CAEBV is 2.5 × 10^4^ copies/ml (range 5.00 × 10^2^ -5.8 × 10^6^ copies/ml) (Table 2). The EBV antibody profile is listed in Table 2. Antibody to EBV VCA IgG was elevated in 21 of 21 patients tested with a conventional assay. Antibody titers to EBV early antigen (EA) were elevated in 8 of 18 patients tested with EBV EA assay. Antibody titers to EBNA were elevated in 7 of 7 patients tested with EBNA assay.

**Table 2:**
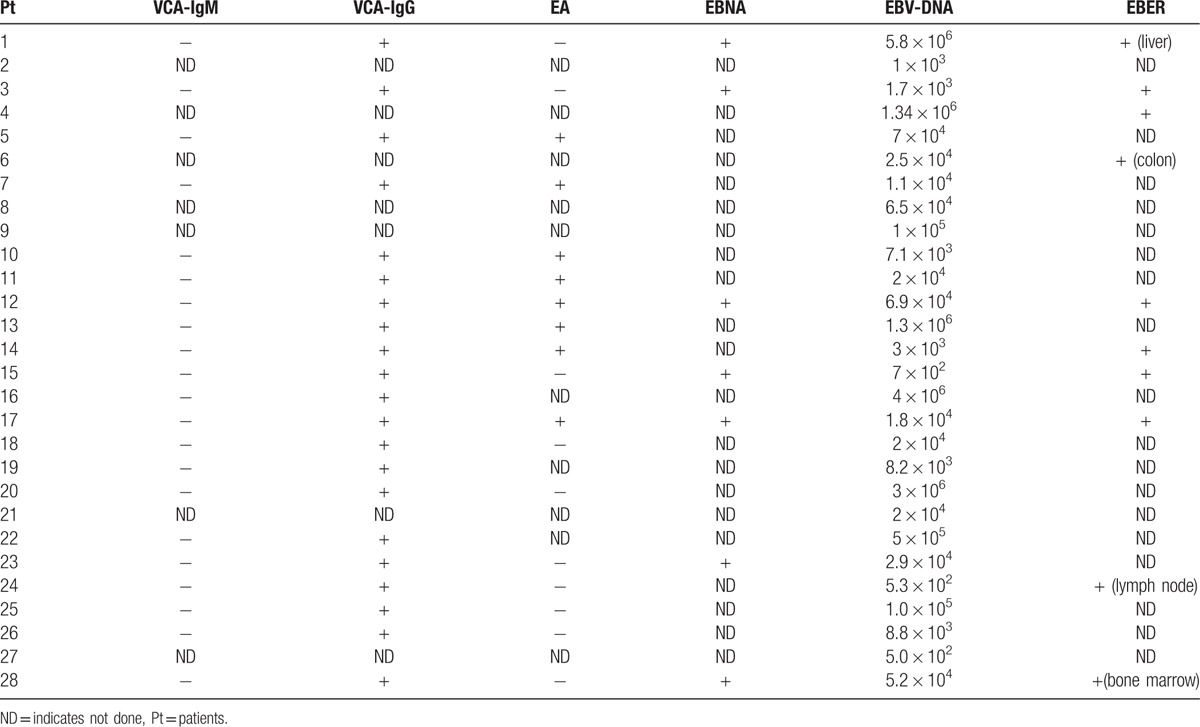
EBV viral profile of CAEBV.

A total of 22 patients had completed peripheral lymphocyte immunophenotype evaluation. A striking features revealed that our patients had significant low level of B cells, NK cell, CD4 count, and CD8 count, even in the absence of chemotherapy. In our patients, 90.9% (20/22) had low numbers of CD19+ B cells (Table 3), and the median of CD19+ B cells was 25 × 10^9^/L. 68.2% (15/22) had low NK cells (Table 3) and the median of NK cell is 69 × 10^9^/L. 77.2% (17/22) had low CD4+ T cells (Table 3), and the median of CD4+ T cell is 311  × 10^9^/L. 54.5% (12/22) had low CD8+ T cell count (Table 3) and the median of CD8+ T cell is 280 × 10^9^/L. The CD4/CD8 ratio was inverted (<1) in 9 patients (32.1%). The levels of expression of activation markers (CD38 and HLA-DR) on CD8+ T cells were significantly increased. The median of the percentage of CD38+CD8+ T cell is 89.3% (range 49.1%–99.7%). The median of the percentage of HLA-DR+CD38+ T cells is 70.6% (range 17.1%–97.5%). The median of the percentage of CD4+CD28+ T cell was within the normal range (median 95.4%) and the median of the percentage of CD8+CD28+ T cell was also with the normal range (median 42.6%).

**Table 3:**
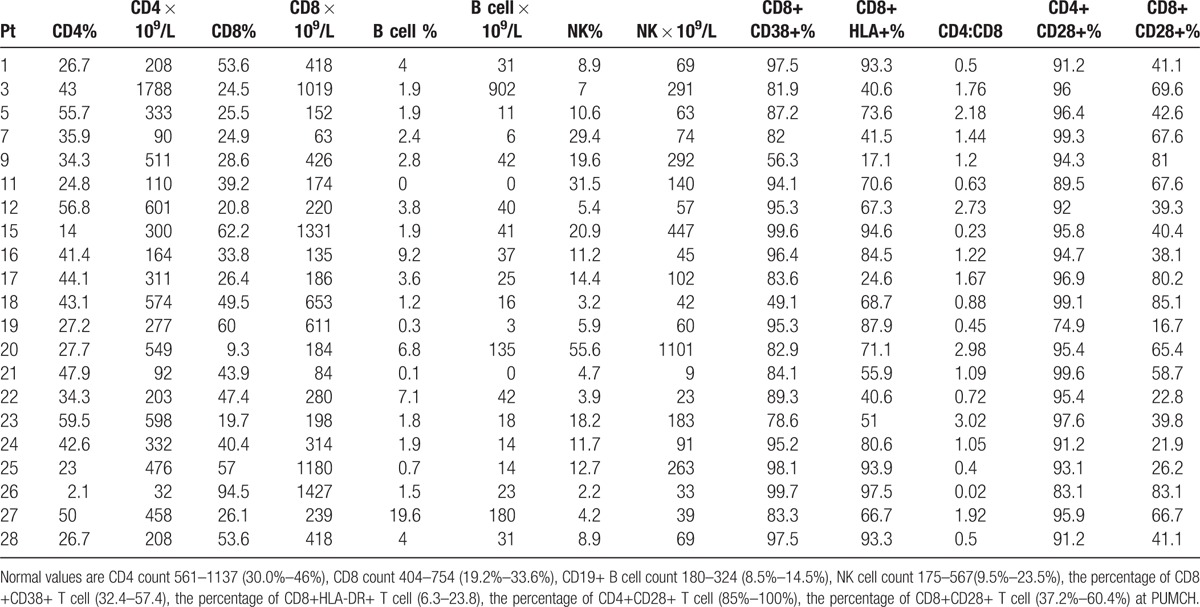
Immunophenotypic data of the CAEBV patients.

In the patients who completed peripheral lymphocyte immunophenotype evaluation, 18.2% (4/22) had low serum IgG level. While most of the patients had low CD19+ B cell count, 13.6% (3/22) had high serum IgG concentration.

## 4 Discussion

In this report, we presented the clinical characteristics and the immunophenotypic features of peripheral lymphocytes in 28 adult onset CAEBV patients diagnosed in PUMCH. To our knowledge, this is the largest series of adult onset CAEBV from China.

In this study, the clinical characteristics of the patients have several important differences from previously reported cases. First, in the largest review of patients with CAEBV, which included 82 patients from Japan, the mean age at disease onset was 11.3 years,^[6]^ whereas the median age at admission in our study was 45 years old. Second, the features of cardiovascular diseases in our study were different from that pediatric cases. Ishihara et al. reported most of the pediatric CAEBV cases with cardiovascular disease included coronary artery aneurysm or myocarditis.^[12]^ In our study on adult onset CAEBV, the clinical manifestations of cardiovascular disease included PAH, decreased cardiac function, and aorta vasculitis. Third, interstitial pneumonitis was more common in Chinese adult onset CAEBV patients (25%). In the largest review of CAEBV patients from Japan, the percentage of patients with interstitial pneumonitis was 4.8%. Fourth, hypersensitivity to mosquito bites, a finding usually associated with NK cell CAEBV, was much less commonly reported in Japan.^[6]^

CAEBV has been thought to be linked to viral replication. Virus loads in serum were analyzed for the 28 CAEBV patients in this study. The results showed noticeable increases in viral loads in all the CAEBV patients, suggesting that quantitative PCR analysis of serum for EBV were valuable diagnostic methods in CAEBV. Based on these findings, we recommend that if a patient presented with a series of symptoms which fit in the spectrum of symptoms of CAEBV, EBV DNA should be measured.^[13,14]^

The pathogenesis of CAEBV remains unknown. A possible pathophysiology of this disease could be that the imbalance between immunological function and viral pathogenesis. We have known that EBV-infected NK cells and T cells have been found to play a central role.^[3,15]^ However, the study on the assessment of the immnuophentypic features of peripheral lymphocyte of adult onset CAEBV is very limited.^[5–7]^

The abnormalities of immunophenotype of peripheral lymphocytes in this study are striking. In this study, 86.3% of the CAEBV patients had low numbers of CD19+ B cells, 68.2% had low NK cells, 77.2% had low CD4+ T cells and 59.1% had low CD8+ T cell. 32.1% of the CAEBV patients had inverted CD4/CD8 ratio. These results suggest that abnormalities in the immunophentype of peripheral lymphocyte subsets may be associated with the development of CAEBV.

In this study, the percentage of low CD19+ B cell count is much higher than that reported in Japan. We found that 90.9% had low CD19+ B cell count. In Cohen’ study, which included 19 CAEBV patients (mean age 19, range 4–51), 43% had low levels of B cells in the absence of rituximab.^[7]^ In this study, the percentage of low NK cells is much higher than that other reports. In our patients, 68.2% had low NK cells. Cohen et al. found that approximately 33% of of CAEBV in the United States had low NK-cell numbers.^[7]^ In a study of 81 patients with CAEBV in Japan, none was reported to have low NK-cell numbers.^[6]^ Lu Gen et al. reported 34.8% of the Chinese pediatric CAEBV patients had low NK cell counts.^[16]^ While most of the patients had decreased NK cell, 4.5% (1/22) of the patients had elevated NK cell. The percentage of elevated NK cell is much lower than other reports. In a study by Kimura et al., 19% (15/81) had elevated NK-cell.^[17]^ In this study, the percentage of low CD4+ T cell was much higher than that other reports. In this study, the percentage of low CD4+ T cell was 77.2%. Cohen et al. found that 38% of of CAEBV in the United States had low CD4 cells count.^[6]^ While the percentage of decreased level of B cells in our patients was much higher than Cohen's study, the percentage of decreased level of serum IgG level was much lower. The percentage of decreased level of serum IgG level was only 18.2%, while 42% of the patients that reported in the United States developed hypogammaglobulinemia.^[7]^

This study has several limitations. First, it consists of a relatively small sample size. Adult onset CAEBV cases are relatively rare. As such, our findings may not be necessarily generalizable to other populations. Second, it is a retrospective study and therefore do not provide information regarding changes in immunophenotype of peripheral lymphocytes.

## 5 Conclusions

There is difference of clinical manifestation characteristics between Chinese adult onset CAEBV patients and other reported CAEBV cases. Our study showed that there was an imbalance in lymphocyte subsets and disturbance in cellular immunity in CAEBV patients. The percentages low level of B cells, NK cells, and CD4+ T cells are much higher than reported.
